# Prevalence, risk factors and adverse perinatal outcomes for Chinese women with intrahepatic cholestasis of pregnancy: a large cross-sectional retrospective study

**DOI:** 10.1080/07853890.2022.2136400

**Published:** 2022-10-22

**Authors:** Kaiqi Wu, Binin Yin, Shuai Li, Xiaojun Zhu, Bo Zhu

**Affiliations:** a Department of Clinical Laboratory, Women’s Hospital, School of Medicine, Zhejiang University, Hangzhou, China; b Department of Obstetrics, Women’s Hospital, School of Medicine, Zhejiang University, Hangzhou, China

**Keywords:** Intrahepatic cholestasis of pregnancy, prevalence, risk factors, perinatal outcomes

## Abstract

**Background:**

Intrahepatic cholestasis of pregnancy (ICP) is the most common pregnancy-related liver disorder and may cause adverse perinatal outcomes. This large cross-sectional retrospective study aimed to evaluate the prevalence and related risk factors of ICP and determine the adverse perinatal outcomes.

**Methods:**

This large cohort study from 1 January 2018 to 31 December 2019, included 39,742 eligible pregnant women. Data were extracted from the institutional electronic medical record database and analyzed using univariate and multivariate logistic regression models to determine the risk factors and adverse perinatal outcomes of ICP.

**Results:**

The overall prevalence of ICP was 3.81%. It was significantly higher in hepatitis B surface antigen (HBsAg) positive than negative women in all age groups, and in women with pre-pregnancy BMI underweight and obesity aged <25 years and ≥35 years than the other age groups. Multivariate logistic regression models showed an increased risk of ICP associated with maternal age <25 years and ≥35 years, pre-pregnancy underweight and obesity, HBsAg positive status, twin pregnancies, low maternal education, inadequate gestational weight gain, multiparous, *in vitro* fertilization, caesarean section history and the number of abortions ≥2. The presence of ICP was associated with increased risk of maternal outcomes of caesarean section and preterm birth, and neonatal outcomes of low birth weight and neonatal unit admission in singleton and twin pregnancies.

**Conclusion:**

This study identified the prevalence, possible risk factors, and associated adverse perinatal outcomes of ICP, which provides useful information for clinicians to identify, counsel, and provide timely management for women at risk.

**KEY MESSAGES**
- Maternal age <25 and ≥35, pre-pregnancy BMI underweight and obesity, hepatitis B surface antigen-positive status, twin pregnancies, low maternal education, inadequate gestational weight gain, multiparous, *in vitro* fertilization, caesarean section history and the number of abortions ≥2 are associated with an increased risk of ICP.
- Further, pregnancies with ICP are associated with an increased risk of maternal outcomes of caesarean section and preterm birth and neonatal outcomes of low birth weight and neonatal unit admission in singleton and twin pregnancies.

## Introduction

Intrahepatic cholestasis of pregnancy (ICP), the most common pregnancy-associated liver disorder, manifests as new-onset pruritus and elevated serum bile acid, typically in the second and third trimesters of pregnancy and resolves spontaneously after delivery [1,2]. The global incidence of ICP ranges from 0.5% to 5.6%, depending on the geographic and ethnic variation, and it is highest in South America and Northern Europe [3–6]. Recently, ICP has been reported to be associated with adverse maternal and foetal outcomes, with a higher risk of preeclampsia, later hepatobiliary diseases, and gestational diabetes mellitus [7–9]. In addition, ICP may cause preterm birth, foetal asphyxia, meconium-stained amniotic fluid, cardiotocography abnormalities, a low (<7) 5-min Apgar score, respiratory distress syndrome, and even intrauterine foetal death [5,10–13]. The pathogenesis of ICP is multifactorial and remains unclear although hormonal, genetic, and environmental factors have been implicated [4,14–16]. Previous studies have shown that multiple pregnancies, *in vitro* fertilization (IVF), maternal age >35 years, women with a history of liver-related diseases, particularly gallstone and chronic hepatitis C infection, as well as a previous history of ICP increase the risk [8,17–21].

Moreover, a recent study identified maternal age <25 years, pre-pregnancy underweight, and inadequate gestational weight gain (GWG) as risk factors for ICP [22]. However, studies on the risk factors associated with ICP are still few, and most are centred on the effects of ICP on pregnancy outcomes. In this study, we performed a large cross-sectional retrospective analysis to comprehensively explore the prevalence and risk factors of ICP and its influence on perinatal outcomes.

## Materials and methods

### Study population and data collection

**Figure 1.**
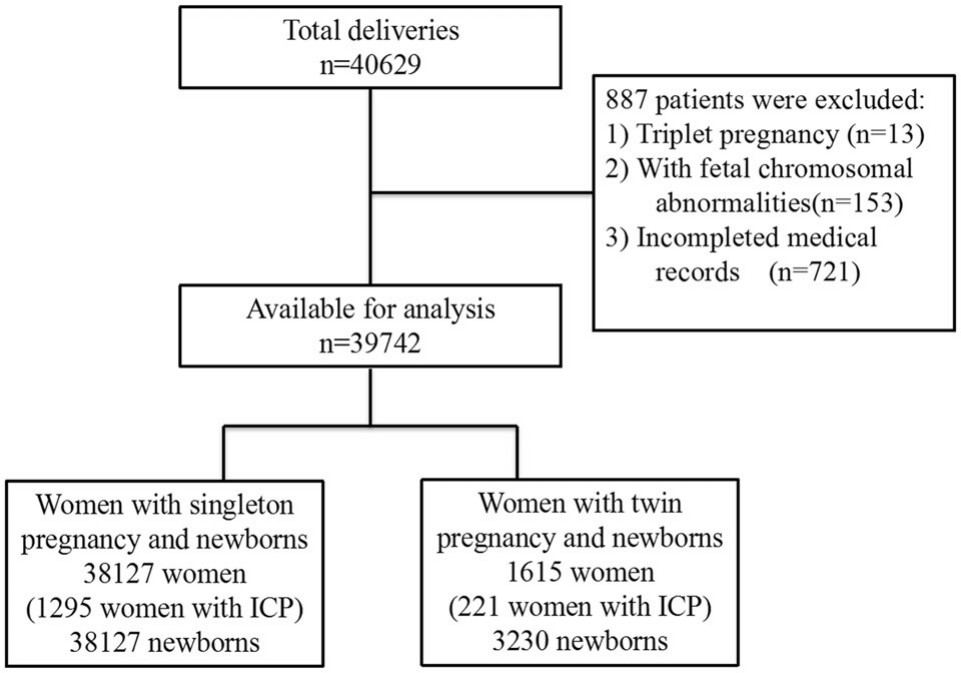
Flowchart of the study population.

This large cross-sectional retrospective cohort study was conducted from 1 January 2018, to 31 December 2019 at the Women’s Hospital, School of Medicine, Zhejiang University, Hangzhou, Zhejiang Province, China. The study protocol was approved by the Ethics Committee of Women’s Hospital, School of Medicine, Zhejiang University (Approval number: IRB-20210200-R). All pregnant women aged 18–55 years who gave birth to a single baby or twins at ≥28 weeks of gestation were included. However, women with foetal chromosomal abnormalities and those without complete medical records were excluded. After excluding 887 women (2.2%), 39,742 women were eventually included in the final analysis. These participants comprised 38,127 women with singleton pregnancies (1293 women with ICP) and 1615 women with twin pregnancies (221 women with ICP) (Figure 1). Maternal demographic characteristics, medical and obstetric history, maternal and infant outcome information were extracted from the institutional electronic medical record database.

### Diagnostic criteria of ICP

In this study, ICP was diagnosed based on the Guidelines for the Diagnosis and Treatment of ICP (2015) from the Department of Obstetrics and Gynaecology, Chinese Medical Association [23]. Accordingly, ICP was defined as follows: (1) unexplained pruritus occurring during pregnancy and (2) unexplained abnormal liver function and/or serum total bile acid (TBA) ≥10 µmol/L in pregnant women. The above conditions will get resolved after delivery in most pregnant women.

### Definitions of demographic and clinical characteristics

The following World Health Organisation (WHO) classification for pre-pregnancy body mass index (ppBMI) was used: underweight (ppBMI < 18.5 kg/m^2^), normal weight (ppBMI: 18.5–24.9 kg/m^2^), overweight (ppBMI: 25–29.9 kg/m^2^), or obesity (ppBMI ≥ 30 kg/m^2^). Maternal education level was categorized as low (either primary education or no education received), medium (secondary or high school education), and high (college/university or higher education). Occupational physical activity levels were grouped into three categories: (1) light (mostly sitting for office work, e.g. secretary), (2) moderate (standing and walking, e.g. store assistant, light industrial worker), and (3) active (walking, lifting, and heavy manual labour, e.g. industrial, building, or farm work). GWG was stratified into the following three categories according to the Institute of Medicine (IOM) guidelines [24]: inadequate, adequate, and excessive. IOM recommended adequate GWG are as follows: 12.5–18 kg for ppBMI <18.5 kg/m^2^, 11.5–16 kg for ppBMI 18.5–24.9 kg/m^2^, 7–11.5 kg for ppBMI 25–29.9 kg/m^2^ and 5–9 kg for ppBMI ≥30 kg/m^2^.

### Perinatal outcomes

The data on adverse pregnancy outcomes were obtained from clinical records. The investigated perinatal outcomes included maternal outcomes consisting of caesarean section, premature rupture of membrane (PROM), preterm birth (delivery before 37 weeks of gestation), abruptio placentae, meconium amniotic fluid, and postpartum haemorrhage; and neonatal outcomes consisting of stillbirth, macrosomia (birth weight ≥ 4000 g), low birth weight (LBW, birth weight < 2500 g), foetal distress, neonatal asphyxia, and neonatal unit admission.

### Statistical analysis

Demographic and clinical characteristics were reported following a descriptive analysis. Continuous data with normal and non-normal distributions were described as means ± standard deviation (SD) and median interquartile range (IQR), and these variables were analyzed using the Student’s *t*-test and Mann–Whitney *U*-test, respectively. Categorical variables were expressed as numbers or percentages, and Pearson’s chi-square (*χ*^2^) test or Fisher’s exact test was used to assess categorical variables. The stepwise (Wald) method was used for the multivariate logistic regression analysis. Crude and adjusted odds risks (ORs and aORs, respectively) of ICP with 95% confidence intervals (CI) were calculated using multiple logistic regression models. A two-tailed *p-*value < 0.05 or a 95% CI was considered statistically significant and the data were analyzed using the statistical package for the social sciences (SPSS) 23.0 (IBM Corp., Armonk, NY).

## Results

### Demographic and clinical characteristics of the study population

The demographic and clinical data of women with (*n* = 1516) and without (*n* = 38,226) ICP are shown in Table 1. The overall incidence of ICP was 3.81%. Compared with women without ICP, those with ICP showed no difference in mean age (*p* = 0.52); However, they had a higher proportion of patients aged <25 years and ≥35 years (*p* < 0.01), and a higher percentage of those with pre-pregnancy underweight and obesity.

**Table 1.** Demographic and clinical characteristics of the study population according to intrahepatic cholestasis of pregnancy.

	ICP (1516)	Non-ICP (38,226)	*p* Value
Maternal age, mean (SD), years	31.20 ± 4.48	31.13 ± 4.41	0.52
Maternal age category [*n* (%)], years			<0.01
<25	114 (7.52)	1751 (4.58)	
25–34	1008 (66.49)	27,892 (72.97)	
≥35	394 (25.99)	8583 (22.45)	
Pre-pregnancy BMI [*n* (%)] (kg/m^2^)			0.01
Underweight (<18.5)	290 (19.13)	6563 (17.17)	
Normal weight (18.5–24.9)	957 (63.13)	26,115 (68.33)	
Overweight (25.0–29.9)	88 (5.80)	2458 (6.43)	
Obesity (≥30)	19 (1.25)	233 (0.61)	
Data missing	162 (10.69)	2857 (7.47)	
Maternal education [*n* (%)]			<0.01
Low	150 (9.89)	2378 (6.22)	
Medium	182 (12.01)	3927 (10.27)	
High	1151 (75.92)	31,079 (81.30)	
Data missing	33 (2.18)	842 (2.21)	
Occupational physical activity [*n* (%)]			<0.01
Light	877 (57.85)	24,225 (63.37)	
Moderate	352 (23.22)	7255 (18.98)	
Active	254 (16.75)	5934 (15.52)	
Data missing	33 (2.18)	852 (2.23)	
Gestational weight gain [*n* (%)]			<0.01
Inadequate	456 (30.08)	9319 (24.38)	
Adequate	523 (34.50)	15,322 (40.08)	
Excessive	338 (22.30)	10,109 (26.44)	
Data missing	199 (13.12)	3476 (9.10)	
Parity [*n* (%)]			<0.01
Primiparous	528 (34.83)	15,579 (40.75)	
Multiparous	988 (65.17)	22,687 (59.25)	
Twin pregnancies [*n* (%)]			<0.01
No	1295 (85.42)	36,832 (96.35)	
Yes	221 (14.58)	1394 (3.65)	
IVF [*n* (%)]			<0.01
No	1323 (87.27)	35,224 (92.15)	
Yes	193 (12.73)	3002 (7.85)	
Caesarean history [*n* (%)]			<0.01
No	575 (37.93)	22,146 (57.93)	
Yes	941 (62.07)	16,140 (42.22)	
Abortion history [*n* (%)]			0.01
0	760 (50.13)	20,792 (54.39)	
1	426 (28.10)	10,286 (26.91)	
≥2	330 (21.77)	7148 (18.70)	
HBsAg [*n* (%)]			<0.01
Negative	1304 (86.02)	36,261 (94.86)	
Positive	212 (13.98)	1965 (5.14)	
GDM [*n* (%)]			0.18
No	1210 (79.82)	31,053 (81.24)	
Yes	306 (20.18)	7173 (18.76)	
HDP [*n* (%)]			
No	1451 (95.71)	37,253 (97.45)	<0.01
Yes	65 (4.29)	973 (2.55)	
Laboratory measurements			
TBA, median (range) (μmol/L)	24.00 (11.00–264.00)	2.00 (0.00–9.00)	<0.01
ALT, median (IQR) (U/L)	81 (56–215)	31 (24–46)	<0.01
AST, median (IQR) (U/L)	73 (46–183)	21 (15–28)	<0.01

Two independent sample *t*-tests were used for normally distributed variables; Pearson’s Chi-square test or Fisher’s exact test were used for categorical variables; The differences of non-normally distributed parameters were analyzed using Mann–Whitney *U*-test.

ICP, intrahepatic cholestasis pregnancy; SD, standard deviation; BMI, body mass index; IVF, *in vitro* fertilization; GDM, gestational diabetes mellitus; HDP, hypertensive disorders of pregnancy; IQR, interquartile range; TBA, total bile acid; ALT, Alanine transaminase; AST, Aspartate transaminase.

Women with ICP were more likely to be hepatitis B surface antigen (HBsAg)-positive; additionally, they were more likely to have active occupational physical activity, inadequate GWG, a high percentage of multiparity, more twin pregnancies, more IVF, a lower level of education, a higher percentage of the history of caesarean section and abortion, and hypertensive disorders in pregnancy (HDP) than those without ICP. Furthermore, the levels of TBA, aspartate transaminase (ALT), and alanine transaminase (AST) were significantly higher in women with ICP than in women without ICP (All *p* < 0.01).

### Incidence of ICP by age and HBsAg status/ppBMI

**Figure 2.**
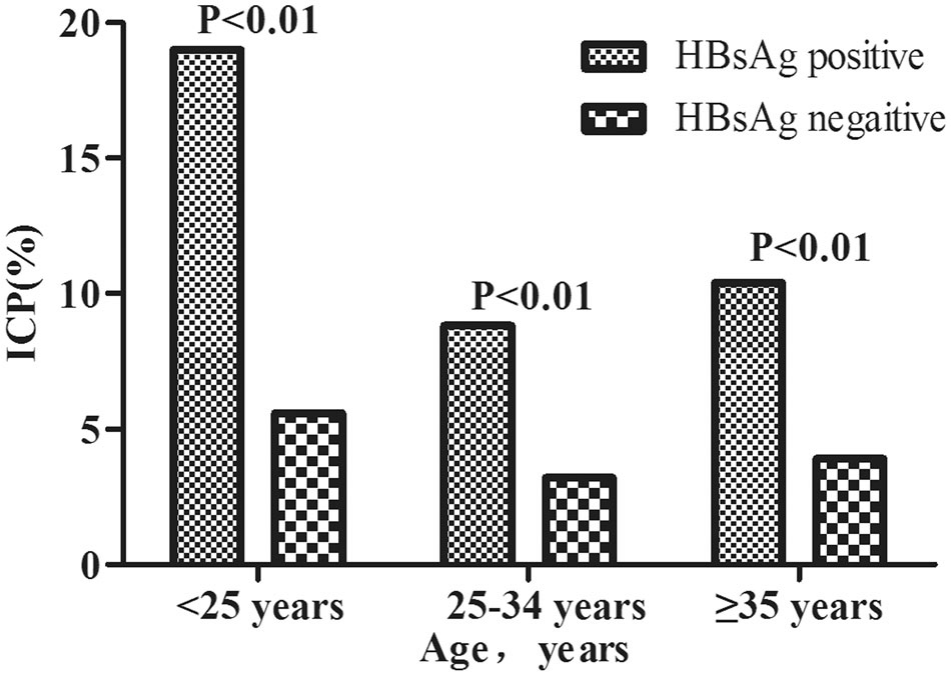
Incidence of intrahepatic cholestasis of pregnancy (ICP) by age and hepatitis B surface antigen (HBsAg) status.

**Figure 3.**
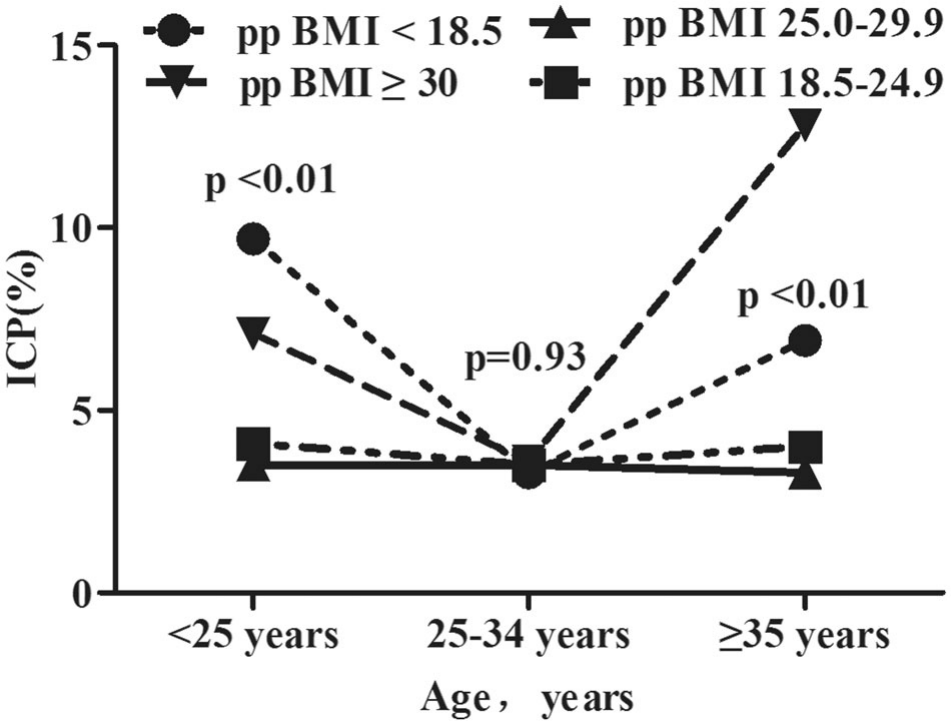
Incidence of intrahepatic cholestasis of pregnancy (ICP) by age and pre-pregnancy body mass index (ppBMI).

Figure 2 shows that the incidence of ICP was significantly higher in women who were HBsAg positive than that in women who were HBsAg negative (*p* < 0.001), in all three age groups (all *p* < 0.01). We also investigated the incidence of ICP according to age and percentage body mass index (Figure 3). The incidence of ICP stratified by ppBMI (underweight, normal weight, overweight, or obesity) was 9.72%, 4.13%, 3.54% and 7.13% among women aged <25 years; 3.32%, 3.34%, 3.49% and 3.58% among women aged 25–34 years; and 6.91%, 4.03%, 3.34% and 14.8% among women aged ≥35 years, respectively. Women who had pre-pregnancy underweight and obesity had a higher incidence of ICP among those aged <25 years and ≥35 years than among the other two age groups, whereas there was no difference in the incidence of ICP in all ppBMI groups among women aged 25–34 years.

### Risk factors for ICP

Table 2 shows the results of the multivariate logistic regression analysis of the association between demographic characteristics and the risk of ICP. Compared to a maternal age within the range of 25–34 years, age <25 years and >35 years was associated with a higher risk of ICP. Regarding ppBMI, being underweight and obese increased the risk of ICP. Low maternal education, inadequate GWG, multiparity, IVF), history of caesarean section and the number of abortions ≥2 were significantly associated with the risk of ICP (all *p* < 0.05). Furthermore, women with twin pregnancies or an HBsAg-positive status showed an increased risk of ICP.

**Table 2.** Factors associated with the incidence of ICP by multivariate logistic regression models.

	Crude OR	*p* Value	Adjusted OR	*p* Value
Maternal age category [*n* (%)], years^a^				
<25	1.78 (1.46–2.17)	<0.01	1.81 (1.44–2.28)	<0.01
25–34	1.00		1.00	
≥35	1.27 (1.13–1.43)	<0.01	1.23 (1.07–1.42)	0.04
Pre-pregnancy BMI [*n* (%)] (kg/m^2^)^a^				
Underweight (<18.5)	1.20 (1.05–1.38)	0.01	1.27 (1.11–1.46)	0.01
Normal weight (18.5–24.9)	1.00		1.00	
Overweight (25.0–29.9)	0.97 (0.78–1.22)	0.81	0.90 (0.71–1.14)	0.39
Obesity (≥30)	2.12 (1.32–3.39)	<0.01	1.96 (1.20–3.21)	0.01
Maternal education^a^				
Low	1.69 (1.42–2.02)	<0.01	1.46 (1.18–1.80)	<0.01
Medium	1.24 (1.06–1.46)	0.01	1.12 (0.93–1.33)	0.23
High	1.00		1.00	
Occupational physical activity				
Light	1.00		1.00	
Moderate	1.34 (1.18–1.52)	<0.01	1.11 (0.92–1.43)	0.26
Active	1.18 (1.03–1.08)	0.02	1.01 (0.87–1.04)	0.37
Gestational weight gain^a^				
Inadequate	1.43 (1.26–1.63)	<0.01	1.45 (1.27–1.65)	<0.01
Adequate	1.00		1.00	
Excessive	0.98 (0.85–1.13)	0.77	0.91 (0.78–1.04)	0.17
Parity^a^				
Primiparous	1.00		1.00	
Multiparous	1.83 (1.54–1.99)	<0.01	1.53 (1.35–1.73)	<0.01
IVF^a^				
No	1.00		1.00	
Yes	1.70 (1.45–1.98)	<0.01	1.22 (1.03–1.46)	0.02
Caesarean history^a^				
No	1.00		1.00	
Yes	2.55 (2.12–2.87)		2.04 (1.81–2.31)	<0.01
Abortion history^a^				
0	1.00		1.00	
1	1.13 (1.00–1.28)	0.04	1.15 (1.01–1.31)	0.04
≥2	1.26 (1.11–1.44)	<0.01	1.21 (1.03–1.41)	0.02
Twin pregnancies^a^				
No	1.00		1.00	
Yes	4.17 (3.60–4.84)	<0.01	3.14 (2.63–3.73)	<0.01
HBsAg^a^				
Negative	1.00		1.00	
Positive	2.96 (2.54–3.44)	<0.01	2.79 (2.36–3.30)	<0.01

^a^Factors for which the multivariate analyses were adjusted.

ICP, intrahepatic cholestasis pregnancy; BMI, body mass index; IVF, *in vitro* fertilization; OR, odds ratio.

### Associations between perinatal outcomes of singleton pregnancy and ICP

We further evaluated the effect of ICP on the perinatal outcomes of a singleton pregnancy. we compared women who had ICP to those who did not. In multivariate analyses, singleton pregnancies with ICP were associated with a higher risk of maternal outcomes of caesarean section and preterm birth, and neonatal outcomes of low birth weight and neonatal unit admission than those without ICP. No significant differences in the maternal outcomes of abruptio placentae, meconium amniotic fluid, postpartum haemorrhage, and neonatal outcomes of stillbirth, macrosomia, foetal distress, and neonatal asphyxia were found between the groups with and without ICP (Table 3).

**Table 3.** Perinatal outcomes of single pregnancy with respect to ICP.

	ICP (1295)	Non-ICP (36,834)	*p* Value	Crude OR (95%CI)	Adjusted OR (95%CI)
Maternal outcome					
Caesarean section	727 (56.14)	14,704 (39.92)	<0.01	1.93 (1.73–2.16)^a^	1.72 (1.54–1.91)^a^
PROM	78 (6.02)	148 (4.02)	0.01	0.48 (0.40–0.56)^a^	0.77 (0.61–1.23)
Preterm birth	288 (22.24)	3411 (9.26)	<0.01	2.81 (2.45–3.22)^a^	2.56 (2.21–3.02)^a^
Abruptio placentae	21(1.62)	722 (1.35)	0.26	0.78 (0.50–1.21)	0.84 (0.52–1.42)
Meconium amniotic fluid	5 (0.39)	251 (0.68)	0.12	0.45 (0.17–2.22)	0.53 (0.25–2.36)
Postpartum haemorrhage	55 (4.25)	1736 (4.71)	0.44	0.90 (0.68–1.18)	1.18 (0.74–1.38)
Neonatal outcome					
Stillbirth	10 (0.77)	262 (0.71)	0.58	1.01 (0.95–1.18)	1.00 (0.98–1.13)
Macrosomia	47 (3.63)	1817 (4.93)	0.03	0.73 (0.54–0.98)^b^	0.87 (0.74–1.26)
LBW	60 (4.63)	405 (1.10)	<0.01	2.14 (1.47–3.11)^a^	2.06 (1.38–3.04)^a^
Foetal distress	175 (13.51)	5911 (16.05)	0.02	0.82 (0.70–0.96)^b^	1.09 (0.86–1.22)
Neonatal asphyxia	15 (1.16)	343 (0.93)	0.40	1.25 (0.74–2.10)	1.48 (0.87–2.42)
Neonatal unit admission	355 (27.41)	5572 (15.13)	<0.01	2.67 (2.13–3.15)^a^	2.42 (1.99–3.02)^a^

*Note*. Multivariate analyses were adjusted for age, pre-pregnancy BMI, maternal education, gestational weight gain, parity, IVF, caesarean history, abortion history, HBsAg status, HDP. The results were presented with an adjusted odds ratio, aOR (95% CI).

HDP, hypertensive disorders of pregnancy; ICP, intrahepatic cholestasis pregnancy; PROM, premature rupture of the membranes; LBW, low birth weight; OR, odds ratio; *p*-value was calculated by Pearson’s Chi-square or Fisher’s exact test, which were used to compare the proportions of maternal and neonatal outcomes between the two groups.

^a^*p* < 0.01; ^b^*p* < 0.05.

### Associations between perinatal outcomes of twin pregnancy and ICP

As twin pregnancies were associated with ICP, we evaluated the effect of ICP on the perinatal outcomes of twin pregnancies. In multivariate analyses, twin pregnancies with ICP were associated with a higher risk of maternal outcomes of caesarean section and preterm birth, and neonatal outcomes of low birth weight and neonatal unit admission than those without ICP. No significant differences in maternal outcomes of PROM, abruptio placentae, meconium amniotic fluid, postpartum haemorrhage, and the neonatal outcomes of stillbirth, macrosomia, foetal distress, and neonatal asphyxia were found between the groups with and without ICP (Table 4).

**Table 4.** Perinatal outcomes of twin pregnancy with respect to ICP.

	ICP (221 women, 442 newborns)	Non-ICP (1394 women, 2788 newborns)	*p* Value	Crude OR (95%CI)	Adjusted OR (95%CI)
Maternal outcome					
Caesarean section	207 (93.67)	1032 (74.03)	<0.01	2.55 (1.45–4.46)^a^	2.31 (1.24–3.67)^a^
PROM	25 (11.31)	198 (14.20)	0.05	0.65 (0.42–1.00)	0.74 (0.37–0.92)
Preterm birth	166 (75.11)	780 (55.95)	<0.01	1.81 (1.30–2.50)^a^	1.58 (1.22–2.35)^a^
Abruptio placentae	2 (0.90)	36 (2.58)	0.09	0.29 (0.07–1.21)	0.53 (0.11–1.53)
Meconium amniotic fluid	1 (0.45)	1 (0.07)	0.18	6.58 (0.41–12.64)	6.01 (0.78–10.65)
Postpartum haemorrhage	15 (6.79)	109 (7.82)	0.26	0.73 (0.42–1.27)	0.86 (0.56–1.51)
Neonatal outcome					
Stillbirth	1 (0.23)	15 (1.08)	0.12	0.66 (0.39–3.19)	0.89 (0.61–2.14)
Macrosomia	0	0			
LBW	301 (68.10)	1694 (60.76)	<0.01	1.38 (1.11–1.71)^a^	1.22 (1.08–1.59)^a^
Foetal distress	24 (10.86)	186 (6.67)	0.26	0.71 (0.39–1.29)	0.83 (0.54–1.48)
Neonatal asphyxia	12 (5.43)	106 (3.80)	0.33	0.81 (0.42–1.87)	0.92 (0.63–1.78)
Neonatal unit admission	22(49.77)	1157 (41.50)	<0.01	1.74 (1.33–2.08)^a^	1.55 (1.20–1.92)^a^

*Note*. Multivariate analyses were adjusted for pre-pregnancy BMI, gestational weight gain, parity, IVF, caesarean history, abortion history, HBsAg status, HDP. The results were presented with an adjusted odds ratio, aOR (95% CI).

HDP, hypertensive disorders of pregnancy; ICP, intrahepatic cholestasis pregnancy; PROM, premature rupture of the membranes; LBW, low birth weight; OR, odds ratio; *p*-value was calculated by Pearson’s Chi-square or Fisher’s exact test, which were used to compare the proportions of maternal and neonatal outcomes between the two groups.

^a^*p* < 0.01; ^b^*p* < 0.05.

## Discussion

In this retrospective cohort study, we explored the prevalence of ICP and its associated risk factors and relationship with perinatal outcomes. This study included 39,742 women, and the incidence rate of ICP was 3.81%. We found a significant correlation between the incidence of ICP according to age and HBsAg status/ppBMI. We observed that maternal age <25 or ≥35 years, pre-pregnancy underweight and obesity, HBsAg-positive status, twin pregnancies, inadequate GWG, IVF, low maternal education, multiparous, history of caesarean section, and the number of abortions ≥2 were significantly associated with an increased risk of ICP. In addition, our study demonstrated both singleton and twin pregnancies with ICP had a higher risk for the maternal outcomes of caesarean section and preterm birth, and the neonatal outcomes of LBW and neonatal unit admission than those without ICP.

The incidence of ICP varies globally [6]. ICP is common in China, with a recently reported incidence of 1.2–6% [22,25]. It is reported to be between 0.2% and 2% in western countries but varies widely with ethnicity and geographic location, which is most common in South America and Northern Europe [2,5]. Inconsistent incidence of ICP may be due to multiple pregnancies (up to 22% in one study) [26], IVF [27] and liver disease [8]. The incidence of ICP in a previous analysis of a prospective population-based study of 12,200 deliveries in Anhui, China (6.06%) [22] was higher than the incidence observed in our study. The difference in the incidence of ICP might be due to geographic location, ethnicity, and dietary habits. In this study, we found that women who were underweight and those with obesity had a higher incidence of ICP among those aged <25 years and ≥35 years than those with pre-pregnancy normal weight and overweight women, which suggests that the incidence of ICP was significantly correlated with age and BMI.

These factors may explain the difference in the incidence of ICP among various populations. Moreover, our results showed that maternal age ≥35 and <25 years was a risk factor for ICP, which is consistent with the results of previous studies [22,28,29]. Pre-pregnancy underweight was observed as a risk factor for ICP, which is in accordance with recent reports [22]. Additionally, pre-pregnancy obesity was found a risk factor for ICP. However, the mechanism underlying the correlation of ICP incidence with age and pp BMI is still unknown and requires further investigation. Our findings enhance the previous reports and suggest that pre-pregnancy maintenance of an optimal pp BMI and an age of 25–34 years may decrease the risk of developing ICP.

Women with a history of liver-related diseases have been reported to be at a higher risk of ICP. For example, previous studies have reported the association of hepatitis C infection with an increased risk of ICP, and a higher incidence of ICP in hepatitis C virus (HCV)-positive pregnant women than in those who were HCV-negative [8,30–32]. In this study, the incidence of ICP in HBsAg-positive women was significantly higher than that in HBsAg-negative women, and similar results were also found in all three groups stratified by age. Our results showed that HBsAg positivity was associated with a higher risk of ICP. Several recent studies including our previous research have reported a potential association between maternal HBsAg-positive status and the increased risk of adverse pregnancy outcomes, including ICP [33–36].

Moreover, our previous study showed that a high maternal HBV DNA load status in the second trimester of HBsAg-positive pregnant women was associated with a significantly increased risk of ICP [33]. The mechanism underlying the association of HBsAg-positive status with an increased risk of ICP may involve a hepatocellular systemic inflammatory effect that leads to the deterioration of the hepatic function in pregnant women [37,38]. However, the associated mechanisms require further investigation. Our findings suggest that careful screening of HBsAg status during pregnancy may be useful in decreasing the risk of developing ICP.

Our study also found that twin pregnancies and IVF were associated with a higher risk of ICP, which is consistent with previously reported findings [26,27,39]. The association of ICP with twin pregnancies and IVF may be related to the increased levels of hormones in this population, which triggers the accumulation of progesterone metabolites, resulting in disordered hepatocyte bile acid secretion and cholestasis [4,14,26,40]. Moreover, studies have reported that a higher level of oestrogen in twin pregnancies is correlated with an increased risk of ICP [26]. Oestrogen has been reported to cause cholestasis during pregnancy [41]. our results demonstrated that women with ICP during both singleton and twin pregnancies resulted in higher risk

In addition, our results demonstrated that both singleton and twin pregnancies in women with ICP were associated with a higher risk of the maternal outcomes of caesarean section and preterm birth, and the neonatal outcomes of LBW and neonatal unit admission, which was consistent with the results of previous studies [21,42,43]. No relationship was observed between singleton and twin pregnancies in women with ICP and the maternal outcomes of abruptio placentae, meconium amniotic fluid, and postpartum haemorrhage, and the neonatal outcomes of stillbirth, macrosomia, foetal distress and neonatal asphyxia. Nevertheless, other studies have shown the correlation of ICP with a higher risk of meconium staining of amniotic fluid, stillbirth, foetal distress, and neonatal respiratory distress [12,44–46]. This inconsistency in previous findings could be due to differences in clinical pregnancy management, TBA concentration, and the confounding variables of the studies, which may affect the subsequent risk of stillbirth, foetal distress, and neonatal asphyxia [11].

Our study had some notable limitations. First, we used a retrospective design; therefore, we lacked comprehensive information, such as details of a prior or family history of ICP, which prevented the analysis of genetic factors associated with ICP. Second, the study was limited to only one hospital and the height and pre-pregnancy weight of the included participants were self-reported, which may have resulted in bias. Nevertheless, this study generated salient findings. Based on a large population cohort, our study comprehensively assessed the related risk factors of ICP using multivariable logistic regression analysis to ensure reliable assessments. We found that HBsAg-positive status, age, and ppBMI were significantly associated with ICP, which provides a new perspective and useful information to aid clinicians in identifying and counselling women at risk for ICP. In conclusion, our findings could contribute to developing time management strategies for ICP, which may facilitate further research and improve public health.

## Conclusion

Our study showed that maternal age <25 or ≥35 years, pre-pregnancy underweight and obesity, HBsAg positive status, twin pregnancies, inadequate GWG, IVF, low maternal education, multiparous, caesarean history and the number of abortions ≥2 were significantly related to an increased risk of ICP. Furthermore, the findings imply that women at an optimal ppBMI or age within 25–34 years, and careful screening for HBsAg status during pregnancy may decrease the risk of developing ICP. Additionally, the presence of ICP significantly was associated with increased risk of maternal outcomes of caesarean section and preterm birth, and the neonatal outcomes of LBW and neonatal unit admission. The present findings provide a new perspective and useful evidence for clinicians to identify, counsel and provide timely management for women at risk for ICP. We believe that our findings may facilitate further research to improve public health.

## Author contributions

KW was involved in the conception design, analysis and interpretation of the data, and drafting of the manuscript. BY and SL collected and analyzed the data and assisted in manuscript drafting. XZ collected and analyzed the data. BZ developed the conception design, analyzed and interpreted the data, and revised the manuscript critically for intellectual content.

## Data Availability

The data that support the findings of this study are available from the corresponding author upon reasonable request. The data are not publicly available due to privacy or ethical restrictions.
